# Revolutionizing Heart Transplantation: A Multidisciplinary Approach to Xenotransplantation, Immunosuppression, Regenerative Medicine, Artificial Intelligence, and Economic Sustainability

**DOI:** 10.7759/cureus.46176

**Published:** 2023-09-29

**Authors:** Yousaf Tanveer, Aleena Arif, Tamar Tsenteradze, Nabila N Anika, Danyal Bakht, Quratulain Fatima Masood, Maryam Affaf, Wajiha Batool, Indresh Yadav, Rayan W Gasim, Youssef Mohamed, Mohamed Abdelmonim Khogali Mohamed, Chukwuyem Ekhator, Syed Naveed Mohsin, Rehman Khan

**Affiliations:** 1 Urology, Craigavon Area Hospital, Craigavon, GBR; 2 Internal Medicine, Allama Iqbal Medical College, Lahore, PAK; 3 General Surgery, Cardiology, and Internal Medicine, Tbilisi State Medical University, Tbilisi, GEO; 4 Surgery, Baylor College of Medicine, Houston, USA; 5 Internal Medicine, Holy Family Red Crescent Medical College and Hospital, Dhaka, BGD; 6 Medicine and Surgery, Mayo Hospital, Lahore, PAK; 7 Surgery, National University of Science and Technology, Rawalpindi, PAK; 8 Internal Medicine, Women Medical and Dental College, Abbottabad, PAK; 9 Internal Medicine, Army Medical College, Rawalpindi, PAK; 10 Internal Medicine, Samar Hospital and Research Center Pvt. Ltd., Janakpur, NPL; 11 Internal Medicine, Community Based Medical College Bangladesh, Mymensingh, BGD; 12 Internal Medicine, University of Khartoum, Khartoum, SDN; 13 Intensive Care Unit, Ibrahim Malik Teaching Hospital, Khartoum, SDN; 14 Cardiac Critical Care, King Abdulaziz Medical City, Riyadh, SAU; 15 Neuro-Oncology, New York Institute of Technology, College of Osteopathic Medicine, New York, USA; 16 Orthopedics, St. James's Hospital, Dublin, IRL; 17 General Surgery, Cavan General Hospital, Cavan, IRL; 18 Internal Medicine, Mayo Hospital, Lahore, PAK

**Keywords:** machine learning, cardiac regeneration, stem cell therapy, immunosuppressive therapies, heart transplantation

## Abstract

Heart transplantation (HTx) stands as a life-saving intervention for patients with end-stage heart disease, but the field is fraught with numerous challenges that span from the scarcity of donor organs to long-term complications arising from immunosuppressive therapies. This comprehensive review article offers an in-depth exploration of the multifaceted aspects of HTx. The review covers groundbreaking advancements in xenotransplantation, enabled by cutting-edge genetic engineering techniques, and the promising role of stem cell therapies, particularly porcine mesenchymal stem cells, in cardiac regeneration. It also delves into the evolution and limitations of immunosuppressive therapies and the revolutionary potential of artificial intelligence (AI) and machine learning (ML) in enhancing donor-recipient matching and predicting patient outcomes. Economic considerations, especially in the context of rising healthcare costs, are examined to assess the sustainability of these advancements. The article further discusses the significant improvements in patient outcomes over the years, while highlighting persisting challenges, such as graft failure, rejection, and infection. It underscores the importance of experience and specialized training, evidenced by the presence of an institutional learning curve. The review concludes by advocating for a multifaceted, collaborative approach involving clinicians, researchers, and policymakers to overcome existing challenges. Through coordinated efforts that consider medical, ethical, and economic factors, the field of HTx is poised for further evolution, offering renewed hope for improved patient care and outcomes.

## Introduction and background

The field of heart transplantation (HTx) has undergone a transformative evolution in medical research. Originating from rudimentary experimental beginnings, it has now become a medical milestone, offering a pivotal therapeutic option for individuals suffering from end-stage heart disease [1]. This remarkable journey began in the historic year of 1967 with the first successful human-to-human heart transplant, marking a significant leap in medical science. However, the widespread acceptance of HTx was not immediate. It was not until the 1980s that it became the preferred treatment for patients with end-stage heart failure (HF), largely due to the introduction of cyclosporine (CyA). This breakthrough significantly improved patient survival rates, which rose from a modest 30% between 1967 and 1973 to an encouraging 60% between 1974 and 1980. In the current era, this rate has soared to an impressive 90% [2,3].

Despite these advancements, the field of HTx continues to face substantial challenges that hinder its widespread application. Among the most pressing issues is the chronic shortage of donor organs, which severely limits the number of patients who can benefit from this life-saving procedure. This scarcity has led to a disheartening gap between supply and demand. Additional long-term threats to patient survival include cardiac allograft vasculopathy (CAV) and malignancy. The use of immunosuppressive drugs, although essential for preventing rejection, often results in complications, such as diabetes mellitus, kidney disease, hypertension, and obesity, contributing to patient morbidity and mortality [4,5].

The global volume of adult heart transplants has stagnated at approximately 4,000 per year, despite a nearly 20% increase in the number of adults on the waiting list [3]. Moreover, the five-year survival rate remains at about 70%, with a median survival duration of just 11 years and an annual attrition rate of 3-4%. These statistics have shown little change over the past three decades [2,3].

To address the critical issue of organ scarcity, xenotransplantation has emerged as a promising avenue. This innovative approach involves the transfer of living cells, tissues, or organs between different species. Initially, non-human primates (NHPs) were the focus, but pigs have since become the preferred subjects for xenotransplantation research due to their physiological similarities to humans, rapid reproductive maturity, and lower susceptibility to zoonotic infections. However, the transplantation of porcine organs into humans triggers a robust immune response, leading to rapid rejection [6]. Advances in genetic modification techniques, such as CRISPR-Cas9, aim to overcome these challenges by creating pig organs that closely mimic human organs, thereby reducing the likelihood of rejection [7].

The journey of HTx from its experimental inception to its current status as a life-saving treatment for end-stage heart disease exemplifies the progress made in medical science. However, significant challenges remain, including organ scarcity and complications associated with chronic immunosuppression. Xenotransplantation offers a glimmer of hope in addressing these issues. Through advancements in genetic engineering and immunological strategies, we are on the cusp of revolutionizing the field of transplantation, potentially saving countless lives through the gift of a new heart.

## Review

Exploring the potential and challenges of xenotransplantation

The field of xenotransplantation, involving the transfer of living cells, tissues, or organs between different species, has gained momentum in medical research. This surge in interest is driven by the urgent need to address the severe shortage of human donor organs and tissues. As the demand for life-saving organ transplants continues to rise, the limitations of relying solely on human donors have become increasingly apparent, prompting the exploration of alternative sources. Among the various options, pigs have emerged as a particularly promising donor species due to their anatomical and physiological similarities to humans and their reproductive capabilities and cost-effectiveness [8].

However, the journey toward successful pig-to-human organ transplantation is fraught with complex challenges. These include the activation of both innate and adaptive immune systems, coagulation dysregulation, and the potent influence of inflammation. The process is initiated by a rapid innate immune response, involving natural anti-pig antibodies, complement systems, and cellular responses from neutrophils, monocytes, macrophages, and natural killer (NK) cells. This is followed by an adaptive immune response, primarily characterized by antibody production from B cells [9].

A significant immunological hurdle is the presence of natural anti-pig antibodies in humans and NHPs. These antibodies target antigens on the transplanted pig organs, with galactose-α1,3-galactose (Gal) being the most prominent antigen [10]. This interaction triggers a complement cascade, leading to rapid graft destruction, a phenomenon known as hyperacute rejection. To circumvent this, researchers have explored the use of pigs lacking the Gal antigen, which has shown promise in preventing early antibody-mediated and cellular rejection when combined with effective immunosuppressive therapy [11]. However, this approach is not without its challenges, including the risk of thrombotic microangiopathy in the graft [9].

Recent advancements in heterotopic cardiac xenotransplantation (hCXTx) offer a glimmer of hope. These successes suggest that cardiac xenotransplantation (CXTx) could potentially address the critical shortage of donor organs. Two primary cardiac transplantation procedures have been explored: orthotopic cardiac xenotransplantation (oCXTx) and intrathoracic heterotopic cardiac xenotransplantation (ITHCXTx) [12].

The ultimate goal of xenotransplantation is to save human lives and alleviate suffering related to organ failure [13]. While certain achievements have gained recognition, xenotransplantation remains largely in the preclinical phase, especially for organs with complex vasculature. Ethical considerations also come into play, including concerns related to animal welfare and human autonomy. From a theological ethics perspective, a complete ban on xenotransplantation is not justifiable. Instead, the practice should be conducted responsibly, preserving human dignity and autonomy while ensuring the ethical treatment of animals [8].

Navigating the complex landscape of immunosuppressive therapies

HTx serves as a representative model that encapsulates the broader landscape of transplantation science, a field that has witnessed remarkable advancements over the past half-century. These advancements range from improvements in surgical techniques to innovations in post-operative care. Some of these developments have already translated into tangible benefits for patients, enhancing survival rates and improving the quality of life post-transplant. However, it is important to note that the pace of progress has not been uniform across all areas of this complex field. Specifically, in the intricate domain of immunosuppressive therapies, advancements appear to have reached a plateau. Despite the strides made in reducing acute graft rejection rates, challenges remain in striking the delicate balance between preventing rejection and minimizing the adverse effects of long-term immunosuppression, such as susceptibility to infections and malignancies [14].

Modern immunosuppressive strategies in HTx are based on several key principles. First, they recognize that the risk of graft rejection is highest shortly after implantation and decreases over time. This understanding has led to the immediate post-operative administration of high levels of immunosuppressive drugs, followed by a gradual reduction over the first year. The ultimate goal is to establish the least effective maintenance regimen that prevents graft rejection while minimizing drug-related complications [15]. Second, these strategies aim to use lower doses of multiple drugs to avoid overlapping toxicities. Lastly, they acknowledge the adverse effects of excessive immunosuppression, such as increased susceptibility to infections and malignancies [16].

Immunosuppressive regimens are categorized into induction, maintenance, and rejection therapies. Induction therapy is crucial for approximately 50% of heart transplant patients and addresses issues, such as antibody sensitization and the risk of hyper-acute rejection [16,17]. Maintenance immunosuppression involves combinations of medications, such as calcineurin inhibitors (CNIs), anti-metabolites, and proliferation signal inhibitors (PSIs). Monitoring for rejection episodes during the first year post-transplantation is routine and may include non-invasive blood gene expression profiling to reduce the need for invasive endomyocardial biopsies [15].

Addressing antibody sensitization remains a significant research focus. Current strategies primarily target circulating antibodies, but concerns about antibody rebound leading to severe rejection have emerged. Research is exploring ways to modulate or deplete immune effector cells, particularly memory B cells. Advances in immunotherapies for malignancy treatment also hold promise for extending the limited lifespan associated with malignancy as a major limitation to long-term survival post-transplant [18].

Significant progress has been made in understanding immune responses, immunosuppression, tolerance, and xenotransplantation. Achieving immune tolerance remains the ultimate goal, and research in xenotransplantation has shown promising outcomes. While progress in immunosuppression has been comparatively slower, the field continues to advance, offering hope for improved patient care and expanding the boundaries of transplantation science.

Unveiling the potential of stem cells in cardiac regeneration

Historically, the adult heart was considered to have limited self-renewal capabilities. However, recent discoveries challenge this notion, revealing a degree of cardiomyocyte turnover. Various cardiac stem cell (CSC) and cardiac progenitor cell (CPC) populations have been identified in the last decade, including cardio-sphere-derived cells (CDCs), stem cell antigen (Sca)-1+ cells, insulin gene enhancer protein (Isl)-1+ cells, and cardiac side population cells [19].

Stem cell therapy holds promise for heart transplants and regeneration, particularly following severe cardiovascular events, such as atherosclerotic plaque rupture and myocardial infarction (MI). These events result in significant tissue damage and loss of cardiomyocytes, initiating a two-phase healing process: the early inflammatory and reparative phases [20]. Stem cells are broadly categorized into embryonic stem cells (ESCs) and adult stem cells. ESCs offer advantages, such as enhanced proliferation and pluripotency, but are less favorable for cell-based therapies due to challenges in isolation, ethical considerations, and tumorigenic potential [21].

Adult tissues are not just passive recipients of transplanted organs; they also harbor their reservoirs of stem cells that play a critical role in tissue repair and regeneration. Among these, induced pluripotent stem cells (iPSCs) stand out for their versatility and lower immunological barriers, making them particularly promising candidates for transplantation therapies. iPSCs can differentiate into various cell types, including cardiomyocytes and cardiac progenitors, which are essential for heart function. This opens up new avenues for treating a range of cardiac disorders, potentially revolutionizing the way we approach heart disease treatment [22].

Early-stage clinical trials involving stem cell transplantation have shown some beneficial effects in cardiac repair. These trials yielded modest results, primarily because they used autologous cells requiring up to three weeks for expansion, delaying their application [23]. To address these challenges, researchers have explored alternative cell sources, such as porcine mesenchymal stem cells (MSCs), which offer several advantages for cell-based therapy [24,25].

Efficient delivery and cell retention are critical factors in stem cell-based therapies for heart disease. Various methods for cell administration have been explored but have yielded inconsistent results due to low cell engraftment and survival rates [26]. Researchers are increasingly combining stem cells with synthetic or natural scaffolds to improve cell delivery and retention. Solid scaffolds, such as biomimetic cardiac patches, and soluble materials, including injectable hydrogels, have shown promise in supporting cardiac regeneration [20].

Leveraging artificial intelligence for optimized donor matching in Hex

Artificial intelligence (AI) is a rapidly evolving field that has far-reaching implications across various sectors, including healthcare. At its core, AI aims to replicate and even enhance human cognitive functions, such as decision-making, by leveraging the power of big data and advanced computational algorithms. Within the umbrella of AI, machine learning (ML) emerges as a specialized subset. Unlike traditional AI systems, which are programmed to perform specific tasks, ML systems are designed to learn from data and improve over time. They analyze extensive datasets to identify patterns, make predictions, and optimize task efficiency, thereby becoming more accurate and effective as they are exposed to more data [27].

Understanding the nuanced differences between AI and ML is not just academic; it is crucial for their effective application in medical settings. For instance, while AI can be used for diagnostic imaging, ML can be employed to predict patient outcomes based on a variety of factors, such as medical history and genetic markers. This distinction is vital for healthcare professionals and researchers who are looking to integrate these technologies into clinical practice. By appreciating the unique capabilities and limitations of AI and ML, they can better tailor these tools to meet specific medical needs, thereby maximizing their utility and impact.

The integration of AI and ML technologies in HTx extends beyond traditional applications, such as predicting mortality and graft failure. These tools offer a comprehensive approach to improving the entire patient journey, from initial assessments to post-transplant care. They assist in evaluating patient candidacy, predicting the benefits of transplantation, and assessing the risks of graft failure and post-transplant mortality [28].

One critical application of AI in HTx is the automation of histopathological evaluations of endomyocardial biopsies, the gold standard for assessing rejection risk. Manual examination of these slides is time-consuming, and AI and ML offer a promising alternative by streamlining the identification process, thereby enabling timely clinical interventions [29].

AI is revolutionizing medicine, offering advantages, such as enhanced efficiency, precision, cost reduction, and reduced physician workloads. In HTx, AI aids in predicting survival rates, identifying waitlist mortality factors, and detecting graft rejection. It also has the potential to improve donor-recipient matching, medication adherence, and lifestyle management, thereby enhancing patient quality of life.

However, the full integration of AI tools into clinical workflows faces several challenges. These include the need for data scientists, data access, clinical expertise, and bias mitigation. In addition, cross-border organ transplantation presents complex challenges, such as illegal organ trafficking, variations in legal frameworks, and logistical hurdles, including travel and visa restrictions [30].

Despite these challenges, AI offers a unique opportunity to enhance organ allocation, donor-recipient matching, and post-transplant survival and management. However, the current lack of primary studies progressing from abstract presentations to manuscript publications indicates a need for more thorough validation before AI's widespread integration into clinical practice [31].

Assessing the economic feasibility of innovations in HF management

HF poses a significant healthcare challenge in the United States, affecting over 5.4 million people and imposing a considerable economic burden. The current annual cost of managing HF in the U.S. healthcare system exceeds $31 billion, a figure projected to rise to an estimated $50 billion by 2030. This escalating financial strain underscores the urgent need for effective strategies to mitigate these costs [32].

Patients with HF face substantial financial challenges, incurring healthcare expenses four times higher over two years compared to those without HF. These costs include various expenditures, such as inpatient care for HF exacerbations, outpatient care for ongoing disease management, and chronic therapy costs [33].

A significant factor contributing to the rising costs of HF management is the advent of novel medical therapies. For example, the introduction of neprilysin inhibitors in 2015 has led to increased healthcare expenses. While these therapies have improved patient outcomes, they also impose a significant financial burden. Medicare beneficiaries, for instance, may face out-of-pocket costs exceeding $1,000 annually for these medications [32].

Given the endorsement of these drugs by clinical guidelines due to their positive impact on patient outcomes, the financial implications for HF management are set to escalate further. This escalation affects not only patients but also society at large, as the overall cost of caring for individuals with HF continues to rise [34].

Economic considerations in HTx also require scrutiny. It is crucial to compare the costs associated with heart transplant procedures to those of conventional treatments. Previous studies indicate that HTx leads to added expenses for each year of extended life. These costs are partially reimbursed for heart transplants and primarily involve immunosuppressive, antibiotic, and antihypertensive medications in the first year post-transplantation [35].

Assessing the cost-effectiveness of HTx compared to alternative treatments is complex. For example, orthotopic HTx (OHT) incurs costs of approximately $97,000 per quality-adjusted life year (QALY) gained, compared to intensive medical therapy (IDMT) in OHT-eligible patients. However, when patients receive bridge-to-transplant left ventricular assist devices (BTT-LVAD), the incremental cost-effectiveness ratio (ICER) substantially increases due to high inpatient hospital costs [36].

Looking ahead, a comprehensive approach will be necessary to address these economic challenges. This approach should include conventional treatments, recipient selection, robust social rehabilitation, quality control databases, coordinated research efforts, follow-up, and essential treatment recommendations.

Comparing patient outcomes: traditional methods vs. innovative approaches

HTx has seen remarkable advancements over the years, enhancing various aspects of patient care, such as candidate selection, surgical techniques, immunosuppressive therapies, and postoperative management [37]. The International Society for Heart and Lung Transplantation (ISHLT) registry has documented over 89,000 heart transplants globally since 1983. However, this figure may be an underestimation, highlighting the persistent challenge of donor organ shortages [38,39].

Survival rates post-HTx have significantly improved. Adult recipients now have a median survival of approximately 10.7 years, with 82% surviving the first year and 69% reaching the five-year mark. Interestingly, women tend to have slightly better outcomes. The highest mortality rates occur within the first six months post-transplant, particularly during the perioperative hospitalization period [40-42]. Overall, the quality of life post-transplantation is excellent, often enabling patients to return to their professions. However, long-term complications, such as malignancy, coronary artery vasculopathy (CAV), and graft failure, can develop [37].

The use of LVADs as a bridge to transplant has notably improved patient prognosis, making it comparable to those on inotropes alone. Conversely, patients on extracorporeal membrane oxygenation (ECMO) support face higher mortality rates at the time of transplant. Several independent risk factors, such as pretransplant use of temporary mechanical circulatory support and mechanical ventilation, impact early survival [43].

An interesting aspect of patient outcomes is the institutional learning curve. Centers with early experience in transplantation often report higher mortality rates for their initial procedures compared to subsequent ones [44]. While the factors contributing to this improvement are not fully understood, a combination of preoperative, perioperative, and postoperative elements likely plays a role [45]. Training in HTx, especially for cardiologists and transplant coordinators, positively impacts mortality rates at new centers. However, the components of an effective training program and its optimal duration remain undefined.

In summary, HTx remains the treatment of choice for patients with advanced HF, offering significant improvements in survival, quality of life, and functional status. Despite the progress in reducing morbidity and mortality, challenges, such as graft failure, rejection, and infection, continue to pose hurdles to achieving better short-term and long-term outcomes.

## Conclusions

The field of HTx has evolved remarkably, serving as a life-saving intervention for end-stage heart disease. Despite challenges, such as donor organ shortages and complications from long-term immunosuppressive therapies, innovations, such as xenotransplantation and stem cell therapy, offer promising solutions. AI and ML are revolutionizing donor-recipient matching and outcome prediction, although they are not without limitations. The economic viability of these advancements is crucial, especially given the rising healthcare costs associated with HF.

This article contributes significantly to the medical literature by providing a comprehensive overview of the current state and future directions in HTx. It emphasizes the need for a multifaceted approach involving researchers, clinicians, and policymakers to overcome existing challenges. By focusing on innovations that consider medical, ethical, and economic aspects, this article sets the stage for future advancements in the field, offering renewed hope for improved patient care and outcomes.
